# Supplementary material for: Learning curve and surgical outcome of robotic assisted colorectal surgery with ERAS program
Source: Sci Rep. 2022 Nov 29;12:20566. doi: 10.1038/s41598-022-24665-w (PMC9709162; doi:10.1038/s41598-022-24665-w)
Supplement: Supplementary file 2 — Supplementary Information 2. [file 41598_2022_24665_MOESM2_ESM.zip › RAL Raw data-Ñ[▒K/049/HS_ñw╝╨░O▒Kñσ.pdf]

正式摘要(電子簽章：0000020896020)

W95 / W95 英文標題 【讀取】憑證

臺中榮民總醫院

出院病歷摘要

轉入號： 轉出號：

[redacted]

(8)流水編號	
(9)入院日期	109/12/30 (2020) 直腸外科 W95 - 15
(10)轉科日期	
(11)出院日期	110/01/04 (2021) 住院天數計 5 日

診斷

- 入院：sigmiod colon cancer
- 出院：1.sigmiod colon cancer s/p robot assisted anterior resection with end-to-end anastomosis and small intestine segmental resection with side-to-side anastomosis on 2020/12/31
  2.vasovagal syncope

主訴

- positive stool occult blood was found

病史

- This 65 years old male patient has past history of syncope suspected vasovagal, BPH. He recieved regular health examination at 烏日林新 and positive stool occult blood was found. Colonoscopy showed tumor at sigmoid colon that maligancy was proved in biopsy. PET was arranged and no distant metastasis was found. Thus, he was admitted for surgical treatment.

體檢發現

- conj lithiasis OD; clear OS
  schirmer 12/6 mm on 2020.12.4
  - General appearance :
    - easy looking , conscious clear , without cardiopulmonary distress
    - Vital signs自動帶入date 體溫(BT) 脈搏(P) 呼吸(R) 血壓(BP) 身高(BH) 體重(BW) BMI
      20201230 36.2℃ 71次/分 16次/分 148/86mmHg 175cm 85.7kg 28.0
  - HEENT :
    - Eyes: no anemic conjunctiva, no icteric sclera, normal EOM
    - ENT: no otorrhea, no rhinorrhea, no erythematous mucosa, no injected tonsil, no deformity
  - Neck: supple, freely movable, trachea midline,no deformity, no engorged jugular vein , no palpable thyroid or lymph node enlargement
  - Chest:
    - Inspection: Grossly normal, symmetric and fully expansion, no accessory muscle used, no operation scar, no spider angiomata, no gynecomastia
    - Palpation: no palpable mass or nodule, no subcutaneous emphysema, no tenderness
    - Ausculation: clear breathing sound
  - Heart:
    - Percussion: normal heart size
    - Ausculation: regular heart beat, normal S1, S2, no gallop, no murmur
  - Abdomen:
    - Inspection: in
      - flat contour, symetrically, no scar, no stria, no dilated veins, no rashes
    - Ausculation: normoactive bowel sound, no abdominal bruits
    - palpation: soft, no muscle guarding or rigidity, no local tenderness, no rebounding pain, no palpable mass, no palpable pulsation
    - Percussion: no shifting dulllness, no hepatomegaly, no splenomagaly
  -

Peripheral pulsation :	CA	BA	RA	FA	PA	PTA	DPA
Right side :	++	++	++	++	++	++	++/+++
Left side :	++	++	++	++	++	++	++/+++

  - Genito-anus: no tenderness or palpable mass lesion or rectal shelf, normal external genitalia, normal anal tone, brown stool

CRS CONDITION

  - CEA: 1.56 ng/ml
  -
    - Lung

手術日期及方法

- 手術日期及方法:20201231 1. Robotic (XI) assisted anterior resection with end-to-end anastomosis and small intestine segmental resection with side-to-side anastomosis.
  2. Intraoperative colonoscopy : (-).
  3. High ligation (+) : At IMA root (Via hemolok). IMV was also ligated via hemolok.
  4. Take down from the splenic flexure : (-)
  5. Robotic endoGIA (60-4.3)*1 + EEA 29 mm (Echelon circular powered stapler) to complete end-to-end anastomosis.
  6. Proximal cut end: 3cm, distal cut end: 9 cm
  7. 11cm jejunum was resected, mesentary of the segment of small intestine was ligated, mesetary defect repair(+)
  8. No drainage tube was inserted
  9. ADHESION BARRIER FILM was placed below the extended wound

住院治療經過

- After admission, operation with robotic assisted anterior resection with end-to-end anastomosis and small intestine segmental resection with side-to-side anastomosis was done on 12/31 as schedule. Under ERAS protocol, he could tolerate feeding and pain control. However, he fell down on 1/3 early morning in restroom and hit on his head. Initial conscious loss, hypotension were noted and recoverred after minutes. Brain CT showed no obvious hemorrhage. Under the stable condition, he was discharged on 1/4.

合併症

- nil

一般檢查

- 累積報告：

DATE	TIME	ALB	TP	BIL.T	BIL.D	ALKP	AST	ALT	LDH	CHOL	TG	HDL-C	LDL	UA	NH3	AMY
1091228	1334					62		24								
1091231	0936		6.1							174	182		92	5.6		
1091231	1411	4.1		0.69			25	24								
1100103	0408														54	

1100103	0409			0.29			21	11								

DATE	TIME	WBC	RBC	HGB	HCT	MCV	PLT	BLST	NEUT	BAND	SEG	LYM	MON	EOS	BAS
1091228	1322	6460	4.68	14.3	43.7	93.4	200		70.0			21.5	3.6	4.0	0.9
1091231	1412	12660		14.7			208		91.5			7.2	0.9	0.1	0.3
1100103	0408	8910		9.4			149		59.0			31.3	4.3	4.6	0.8

DATE	TIME	NA	K	CL	CA	BUN	CREAT	Mg	P	CRP	ESR	PCT	LIPASE	LACTATE
1091228	1334	139	3.9			15	1.11							
1091231	0936									0.079				
1091231	1411	141	4.1			12	1.04							
1100103	0409	138	3.4		7.3	24	0.98	1.9						8.3

DATE	TIME	GLUCOSE	PAB	TRF	ZN	CU	KETONE	CK	CKMB	PT-P	PT-C	APTT-P	APTT-C	TRO-T
1091228	1334	109						88	5					5.90
1100103	0408									10.6	10.3	22.6		
1100103	0409							231	6					6.23

DATE	TIME	STOOL-OB	FAT	RBC	WBC	PARA
1100103	1853	4+				

DATE	TIME	PH	PCO2	PO2	SO2	BEB	HCO3	TCO2	HGB	FIO2
1100103	0408	7.386	42.2	150.4	99.2	-0.4	24.7	26.0		

DATE	TIME	PH	BEB	SCVO2	PO2	HCO3	HGB	PCO2	TCO2
1091228	1333	7.337	1.5	41.8	25.6	28.6		54.5	30.2

- 放射線報告 :**檢驗項目 :CT, BRAIN, STEM & CEREBELLUM 報告日期 :1100103**

主　訴 :
Result:
Finding:
Without contrast enhancement CT scan of brain was performed:
> Multiple artifacts over both middle and posterior cranial fossa with difficulty in evaluating the corresponding region brain condition are noted.
> A focal high attenuation lesion over right high parietal subdural region (Axi:26), clinical significance to be determined. Suggest clinical correlation and follow-up.
> Increased subdural effusions over bilateral frontal regions are noted.
> No definite abnormal attenuation change in cerebral parenchyma and cerebellar hemispheres could be noted.
> No evidence of bony lesions or fractures are noted.
Impression:
As above descriptions

檢驗項目 :CHEST AP VIEW (PORTABLE) 報告日期 :1100102
主　訴 :
Result:
Finding:
> Borderline heart size
> Prominence of bilateral hila
> Slightly increased hazy infiltrates in bilateral lungs
> Right mild C-P angle blunting may be due to pleural effusion or pleural change
> Degenerative change of T-spine with osteophyte formations
> On central venous catheter and EKG monitor
Impression:
As above description.

檢驗項目 :CHEST PA VIEW 報告日期 :1091228
主　訴 :
Result:
Finding:
1. The heart size is within normal limit.
2. Tortuosity and atherosclerotic changes of T-aorta.
3. Mild Increased interstitial infiltrations over bil. lungs.
4. Bil. apical pleural change.
5. DJD changes with marginal spur formation of T-L spine.
Impression:
As above description.

特殊檢查

病理報告

其它

出院時情況

- 3.改門診治療

出院指示

藥名	劑量	劑量單位	用法	頻次	數量	數量單位	現況	天數	單位
AVOdart cap 0.5mg	1	CAP	PO	QD	8	CAP	TH	8	天
Urief tab 4 mg	1	TAB	PO	BID	16	TAB	TH	8	天
Ultracet tab	1	TAB	PO	BIDPRN	16	TAB	TH	8	天

出院計劃

主治醫師_2062F 蔣鋅帆 / 住院醫師_2331F 陳凱祥
正式摘要(電子簽章：000000206960220)

W95 / W95

doctorRank:B
